# Late pregnancy screening for preeclampsia with a urinary point-of-care test for misfolded proteins

**DOI:** 10.1371/journal.pone.0233214

**Published:** 2020-05-20

**Authors:** Xing-Min Li, Xue-Min Liu, Jun Xu, Juan Du, Howard Cuckle

**Affiliations:** 1 Shuwen Biotech Co. Ltd, Deqing, Zhejiang Province, China; 2 Department of Obstetrics and Gynecology, Shengjing Hospital of China Medical University, Shenyang, Liaoning Province, China; 3 Department of Obstetrics and Gynecology, Columbia University Medical Center, New York City, New York, United States of America; University of Mississippi Medical Center, UNITED STATES

## Abstract

The aim was to describe and assess a new late pregnancy point-of-care urinary preeclampsia screening test. Urine samples were collected from a consecutive series of 1,532 pregnant women hospitalized at 20–41 weeks gestation in a Chinese single obstetric unit. A simple disposable Congo red based device was newly developed and employed to prospectively test misfolded proteins in pregnant women’s urine. A total of 140 preeclampsia cases were clinically diagnosed, 101 severe and 87 pre-term. Detection and false positive rates were similar in the training and validation subsets with combined 74% and 3.0%. The detection rate was 83% in severe, 86% in pre-term, 49% and 50% in mild and term cases (*P*<0.0001) respectively. In conclusion, a simple point-of-care urinary test for misfolded proteins can be used to screen for preeclampsia in late pregnancy with very high screening performance. To the best of our knowledge, this is the first study to screen for preeclampsia using Congo red based device in Chinese pregnant population.

## Introduction

Worldwide, 2–8% of pregnant women suffer from preeclampsia, it is a major cause of maternal mortality and accounts for a large proportion of preterm deliveries [1]. Primary screening for preeclampsia in early pregnancy and low dose aspirin prophylaxis can prevent about half of preterm cases (i.e. delivering before 37 weeks gestation)—screening detection rate 77% and prevention by aspirin 62% [2]. Screening in late pregnancy is needed for women not screened earlier, and those not prevented by such screening including term preeclampsia, comprising at least two-thirds of cases. Typically, late pregnancy screening is secondary, carried out on women who present with symptoms of preeclampsia or signs of pregnancy related problems.

One approach is maternal serum screening using markers such as soluble fms-like tyrosine kinase-1 (sFlt-1) and placental growth factor (PlGF) [3]. But sFlt-1/PlGF testing requires specific equipment and is not feasible for screening in areas with limited resources. Serum PlGF and another biomarker serum pregnancy-associated plasma protein A (PAPP-A) have been incorporated into the algorithm of the International Federation of Gynecology and Obstetrics (FIGO) for pre-eclampsia screening in first-trimester with detection rates of preterm and term preeclampsia between 75%-77% and 47%-54% at false-positive rate of 10% [4]. Another is maternal urine screening for misfolded proteins based on their affinity for the Congo red dye (‘congophilia’). Initially, proteomic analysis suggested that preeclampsia is associated with misfolded proteins which are aggregated in the placenta [5]. This was then confirmed by four studies which demonstrated that in late pregnancy maternal urine samples taken at the time of preeclampsia diagnosis there was considerably more congophilia than in unaffected pregnancies [6–9].

One study followed up 28 asymptomatic women at high risk of preeclampsia and found that in seven out of nine who were subsequently shown to have the condition, the increase in congophilia occurred more than 10 weeks before diagnosis [6]. This encouraged the researchers to develop a simple point-of-care device that could be used to screen for preeclampsia. In a study of 346 women presenting to a labour and delivery triage the test had an 82% detection rate and 11% false-positive rate for preeclampsia, a performance exceeding that of serum markers tested concurrently [10].

In the current study a new point-of-care urinary Congo red test which is different from the previous method by using capillary tube-based slow release method is described and evaluated in Chinese women hospitalised in late pregnancy. The screening performance is assessed in relation to the severity of preeclampsia as well as any associations between positive test results and other factors.

## Materials and methods

The protocol of this study was approved and informed consent was waived because of the study using residual sample of urinalysis and involving no more than minimal risk by the Institutional Review Board of Shengjing Hospital of China Medical University, Shenyang, Liaoning Province, China. The study was conducted according to the principles expressed in the Helsinki Declaration. All patient data were fully anonymized before accessing.

A consecutive series of hospitalized pregnant women with age ≥18 and gestational age ≥20 weeks was recruited from the No. 1 Obstetrics Ward of the Shengjing Hospital between May and December 2017. Women with infectious diseases, macroscopic urine color changes or receiving therapy in clinical trials were excluded. 1,532 women were finally included.

Mid-stream urine samples were obtained for testing misfolded proteins. Information was collected at the time of admission on maternal age and gestational age, as well as risk factors for preeclampsia including number of fetuses, family history of preeclampsia, hypertension and diabetic status. Blood pressure and proteinuria were measured, the latter using a dipstick and/or a subsequent 24 hour collection. Investigators were blinded with the misfolded proteins test results.

Congo red bound to misfolded proteins in an aqueous solution migrates differentially on cellulose membrane, forming different dyeing patterns compared with a free Congo red solution. We discovered that the differences are especially apparent when the solution is slowly released into a small area on the cellulose membrane (qualitative fast filter paper) through a fine-tipped capillary tube. The more Congo red is bound to misfolded proteins, the dye spreads more evenly on the membrane and less possible to bind with cellulose. On the basis of this finding, a point-of-care device employing the capillary tube-based slow release method (termed the CapCord test, commercially available from Shuwen Biotech) was designed and manufactured. The device includes a plastic pipette to drop urine to a well containing Congo red (0.1 mg/ml), and a capillary applicator to transfer the mixture to cellulose membrane compartment and slowly released. The test produces a result within 3 minutes.

**Fig 1:**
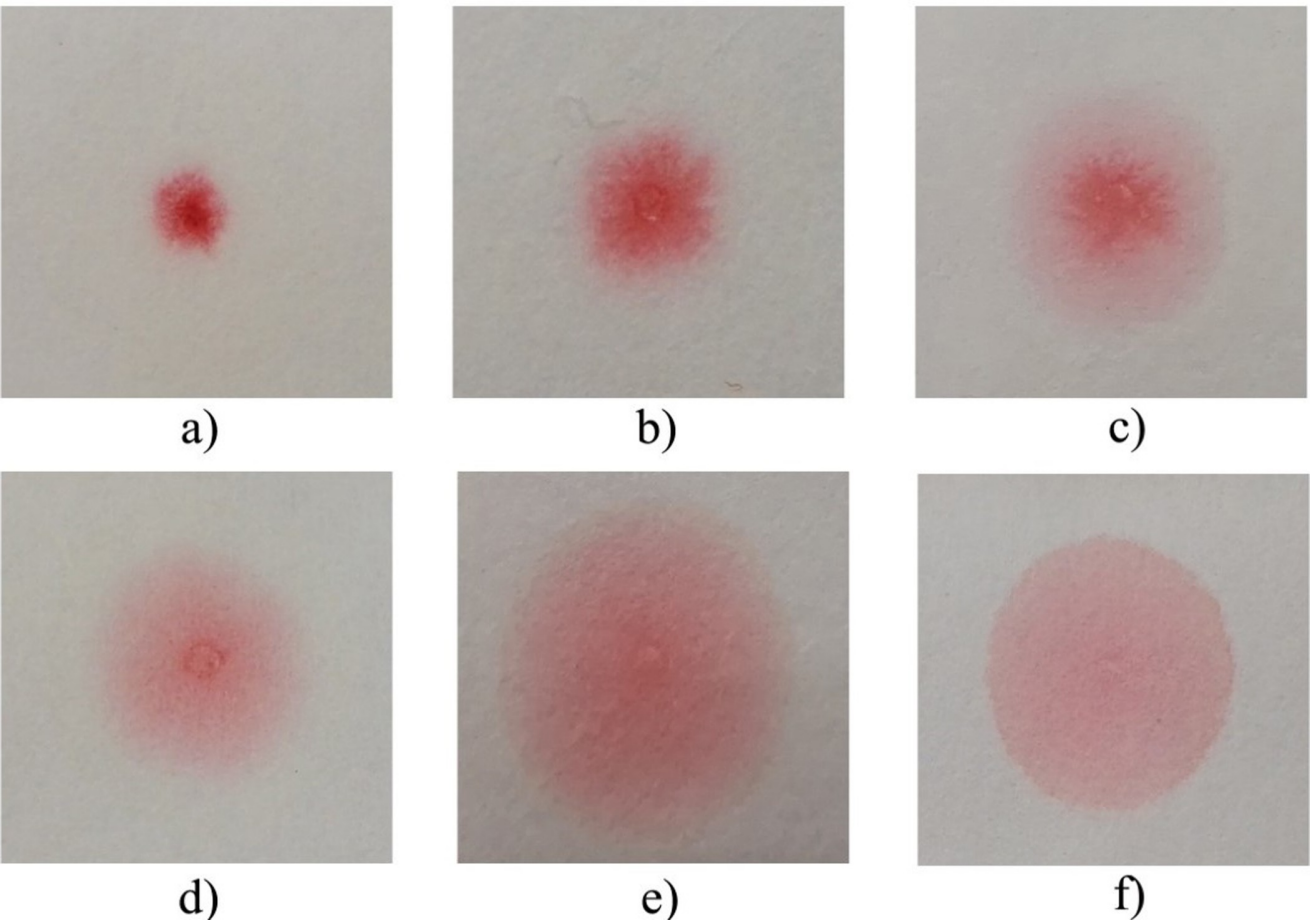
Classification of Congo red staining patterns (a) Small non-diffused red dot; (b) Mildly diffused dot, scarlet pseudopodium; (c) Diffused dot, scarlet pseudopodium, pink penumbra; (d) Small dot, irregular partly diffused pale red penumbra; (e) Red and scarlet dot, partly diffused pale red penumbra; (f) Large uniform pale diffused dot.

All urine samples were tested using this device. Each scorer classified the pattern of the dye into six categories comparison with an illustrative pattern sheet considering the spreading evenness and the tendency of the dye concentrated in the limited central area (Fig 1). Prior to the study, the scorers were trained to achieve standardization avoiding artificial mis-scoring; reproducibility of scoring was assessed at that time. Results were randomized into a ‘training set’ of 770 samples used to group dying pattern categories into positive and negative and compared with the remaining ‘validation set’ of 762 samples. Pregnancies were followed up and diagnosis of preeclampsia and severity was made according to criteria of the American College of Obstetricians and Gynaecologists [11].

The comparison of CapCord with Congo Red Dot (CRD) test was performed in women with enough residual urine samples. CRD test was conducted as per described [6] but using different image analysis. Congo red retention (CRR) rate was calculated by the ratio of gray (Image J software, http://imagej.nih.gov/ij/) after and before wash-up.

SPSS 22.0 (IBM, Armonk, NY) statistical software was used for statistical analysis. Measurement data were expressed as mean ± standard deviation (SD). Statistical analysis was performed between affected and unaffected pregnancies using the Chi-square test; statistical significance was based on *P*<0.05. Comparisons were made according to admission characteristics, preeclampsia risk factors and initial assessments and the detection and false-positive rates according to those which were statistically significant. Nonparametric Spearman's rank correlation was used to assess the association between spread patterns and blood pressure, urinary protein. Receiver operating characteristic (ROC) curves and area under curve (AUC) were used to quantify the performance of biomarker. Z test was used to compare AUC.

## Results

Among the 1,532 consecutive pregnancies, 867 were admitted for symptoms which may or may not be associated with preeclampsia and the most common symptom is bleeding/discharge or abdominal pain 601 (39.2%) followed by elevated blood pressure 95 (6.2%) and fetal status/movement 78 (5.1%) (S1 Table). Asymptomatic cases were admitted for delivery. The mean gestational age at urine sampling is 36.6 weeks (SD = 4.2 weeks). 1,443 (94%) were tested at late pregnancy (≥28 weeks). A total of 140 preeclampsia cases were diagnosed (9.1%), of which 101 were severe and 87 were pre-term, delivering before 37 weeks gestation. Most of the cases were diagnosed on the day of admission and only five were diagnosed subsequently.

Table 1 shows the admission characteristics, preeclampsia risk factors and initial assessments in affected and unaffected pregnancies. As expected, there were highly statistically significant difference between those with preeclampsia and unaffected pregnancies in the gestation at admission, symptoms being included in the reason for admission, specific risk factors for preeclampsia and the initial blood pressure and proteinuria measurements.

**Table 1:** Admission characteristics, preeclampsia risk factors and initial assessments in affected and unaffected pregnancies.

Characteristic*		Preeclampsia (n = 140)	Unaffected (n = 1392)	Statistical significance (*P*)
Admission characteristic				
Preterm gestation (n = 514)		108(77%)	406(29%)	<0.0001
Symptoms (n = 867)		126 (90%)	741 (53%)	<0.0001
Preeclampsia risk factor				
Maternal age over 35 (n = 391)		30 (21%)	361 (26%)	0.24
Multiple pregnancy (n = 30)		8 (5.7%)	22 (1.6%)	<0.001
Previous preeclampsia (n = 137)		86(61%)	51(3.7%)	<0.0001
Hypertension:	Chronic (n = 30)	19 (14%)	11(0.8%)	<0.0001
	Gestational (n = 29)	14 (12%)	15 (1.1%)	<0.0001
Diabetes:	Pre-gestational (n = 22)	9 (6.4%)	13 (0.9%)	<0.0001
	Gestational (n = 203)	30 (23%)	173 (13%)	<0.001
Initial assessment				
Blood pressure raised**(n = 184)		123 (88%)	61 (4.4%)	<0.0001
Proteinuria***(n = 647)		132 (94%)	515(38%)	<0.0001

*excluded from proportions: symptoms–missing indication for admission; gestational hypertension–chronic cases; gestational diabetes–pre-gestational cases.

**systolic ≥140 mmHG or diastolic≥90 mmHG.

***dipstick 1+ or more, or concentration ≥300 mg/24hr.

Table 2 shows the distribution of Congo red dying patterns in affected and unaffected pregnancies included in the training subset. The best discrimination was achieved by classifying the last three patterns (d)-(f) as ‘positive’ and patterns (a)-(c) as ‘negative’. Using that classification the detection and false-positive did not differ significantly between the training and validation subsets (Table 3). Consequently, the best estimate of performance was provided by combining the subsets with a detection rate of 74% (103/140) and false-positive rate of 3.0% (42/1392). The detection rate was significantly higher in severe (83%) or preterm cases (86%) compared with mild (49%) or term (50%) cases (Table 3; *P*<0.0001). According to gestational age at sampling, the detection rate was significantly higher before 28 weeks (*P*<0.05) and before 34 weeks (early-onset) (*P*<0.01). Spread patterns as ranked data were associated with systolic pressure, diastolic pressure and 24 hr urinary protein (*P*<0.0001).

**Table 2:** Training subset: Distribution of Congo red dying patterns in affected and unaffected pregnancies.

Pattern (see Fig 1)		Preeclampsia (n = 66)	Unaffected (n = 704)
(a)	Small non-diffused red dot	2 (0.3%)	225 (32%)
(b)	Mildly diffused dot, scarlet pseudopodium	9 (14%)	392 (56%)
(c)	Diffused dot, scarlet pseudopodium, pink penumbra	5 (7.6%)	65 (9.2%)
(d)	Small dot, irregular partly diffused pale red penumbra	1 (1.5%)	1 (0.1%)
(e)	Red and scarlet dot, partly diffused pale red penumbra	10 (15%)	12 (1.7%)
(f)	Large uniform pale diffused dot	39 (59%)	9 (1.3%)

**Table 3:** Congo red detection and false-positive rates according to scoring subset, type of preeclampsia, characteristics, preeclampsia risk factors and initial assessments.

	Detection rate	Statistical significance (*P*)	False-positive rate	Statistical significance (*P*)
Scoring subset				
Training	76% (50/66)	0.58	3.1% (22/704)	0.81
Validation	72% (53/74)		2.9% (20/688)	
Type of preeclampsia				
Severe	83% (84/101)	<0.0001	-	-
Mild	49% (19/39)			
Delivered pre-term	86%(75/87)	<0.0001	-	-
Term	50% (20/40)			
Late pregnancy	70.2% (87/124)	<0.05	-	-
Mid-pregnancy	100% (16/16)			
Early-onset	86.2% (56/65)	<0.01	-	-
Late-onset	62.7% (47/75)			
Admission characteristic*				
Pre-term gestation	83%(90/108)	<0.0001	3.0% (12/406)	0.93
Term	41%(13/32)		3.0% (30/986)	
Symptoms	75% (95/126)	0.14	2.8% (21/741)	0.67
Asymptomatic	57% (8/14)		3.2% (21/651)	
Preeclampsia risk factor*				
Multiple	88% (7/8)	0.36	14% (3/22)	0.003
Singleton	73% (96/132)		2.8% (39/1370)	
Previous preeclampsia	69%(59/86)	0.09	43%(22/51)	<0.0001
No previous	81%(44/54)		1.5%(20/1341)	
Chronic hypertension	79% (15/19)	0.57	0.0% (0/11)	0.56
None	73% (88/121)		3.0% (42/1381)	
Gestational hypertension	79% (11/14)	0.6	6.7% (1/15)	0.41
None	72% (77/107)		3.0% (41/1366)	
Pre-gestational diabetes	78% (7/9)	0.77	7.7% (1/13)	0.32
None	73% (96/131)		3.0% (41/1379)	
Gestational diabetes	57% (17/30)	<0.05	4.0% (7/173)	0.37
None	78% (79/101)		2.8% (34/1206)	
Initial assessment				
Blood pressure high**	76% (93/123)	0.14	4.9% (3/61)	0.37
Normal	59% (10/17)		2.9% (39/1331)	
Proteinuria***	77% (102/132)	x	6.8%(35/515)	<0.0001
Normal	13% (1/8)		0.7% (6/828)	
Overall	74% (103/140)	-	3.0% (42/1392)	-

*excluded from proportions: symptoms–missing indication for admission; gestational hypertension–chronic cases; gestational diabetes–pre-gestational cases.

**systolic ≥140 mmHG or diastolic≥90 mmHG.

***dipstick 1+ or more, or concentration ≥300 mg/24hr.

Among the 103 Congo red true-positives only one did not have proteinuria at the time of testing and in this case only a dipstick was carried out without subsequent 24 hr urine determination. Of the 42 Congo red false-positives all but seven had proteinuria at the time of testing and the discrepant cases were examined only by dipstick. Three of the false-positives had raised blood pressure at that time, but not later, and for a further nine either systolic was within 10 mmHG or diastolic within 5 mmHG of being classified as hypertension.

Table 3 also compares the detection and false-positive rates according to admission characteristics, preeclampsia risk factors and initial assessments. The detection rate was higher in cases tested preterm, as expected since this group included 91% of the severe and all of the preterm preeclampsia pregnancies. However, the detection rates of severe (88%) and mild preeclampsia (73%) were not statistically significant in preterm preeclampsia cases. There were statistically significant higher false-positive rates in multiple pregnancies and in those with a previous preeclampsia. Whilst there was a significantly lower detection rate in gestational diabetes (*P*<0.05), this is likely to be a chance finding since many statistical comparisons were being made. The highly statistically significant increases in both detection and false-positive rates among women with proteinuria, is an expression of the expected association between misfolded and urinary protein as shown above, but the false positive rate would be 38% assuming urinary protein alone as a preeclampsia biomarker, much higher than that of misfolded protein (3%).

Of the 267 women with enough urine samples to undergo urinary CRD test, 80 were preeclampsia and 55 were severe PE. The performance of CapCord and CRD test in this cohort was similar with AUC 0.77 and 0.74 (*P* = 0.41). When cut-off of CRR was set at 17% with best Youden index, the detection rate and false positive rate of CRD test were 65% and 22.5%. The detection rate and false positive rate of CapCord test were 71.3% and 17.6%. Both methods had better detection rate for severe preeclampsia (CapCord vs. CRD test = 81.8% vs. 76.4%).

## Discussion

This study confirms that a simple point-of-care urinary device for misfolded proteins can be used to screen for preeclampsia in late pregnancy in Chinese population, with a very high performance. The CapCord test is different from previous reported device [10]. The key point of the current device is using fine-tipped capillary tube to slowly release the mixture of urine and Congo red to get different spread patterns which are more distinguishable and reproducible. The overall detection and false-positive rates of 74% and 3.0% respectively compare favourably with rates of 67% and 8.8% in a study using a similar device [10] as well as rates of 80% and 11% using a less stringent interpretation of Congo red patterns in the latter device. The difference in detection rates between the two studies might be due to sample group, but false-positive rate 3.0% might be superior considering the sample size is 4 times more than that of previous study [10]. It is warranted for a further study to perform the both methods simultaneously.

In screening point-of-care devices are generally advantageous compared with laboratory assays due to lower cost, increased speed and less requirement for skilled technicians. Previous studies of Congo red screening for preeclampsia were carried out using the laboratory based ‘retention’ assay developed by Buhimschi and colleagues [6]. The sample was first assayed for total protein and standardised by concentration or dilution to achieve a given protein level. Congo red was added and the mixture spotted onto a nitrocellulose membrane, scanned for optical density, washed with increasing amounts of methanol to remove unbound colour and scanned again. The result was expressed as the percentage of staining retained after the washing step. Sammar and colleagues later showed that scanning and washing were unnecessary because of visual difference in staining pattern between affected and unaffected pregnancies [8]. The current device obviates the need for total protein measurement, already contains Congo red and is based on visually determined patterns achieving similar detection rate with CRD test [6]. The time needed for the test is at most 3 minutes and requires little operator skill.

Using the current device the detection rate was found to be higher in severe (83%) or pre-term cases (86%) compared with 49% and 50% in mild or term cases. The difference of detection rate between severe and mild preeclampsia is consistent with the correlation between spread pattern and blood pressure or urinary protein. The detection rate for pre-term preeclampsia is higher than term cases which may support the theory that these two are distinct phenotypes of preeclampsia [12]. It needs to be addressed in future study. The higher detection rate before 28 weeks and before 34 weeks (early-onset) indicates the usefulness for more intense surveillance by early detection. The other point-of-care study did not break down the results according to severity but this was done in two studies using the laboratory-based retention assay. In one study high retention was reported for 91%, 89% and 75% of those with superimposed, severe and mild preeclampsia respectively [6]. The other study reported the average retention which was 82% in severe and 61% in mild preeclampsia compared with 38% in normotensive controls [9]. As described above, the retention rate might vary in different cohorts and status of preeclampsia, which makes it difficult to set a uniform cut-off. Although previously urinary congophilia for the detection of preeclampsia has been studied in Caucasians [7], Indians [9] and Mexicans [13], this is the first urinary congophilia study of screening for preeclampsia in Chinese population.

In this paper several factors whose incidence differed in preeclampsia cases were considered. For example, about three times as many pregnancies with preeclampsia were admitted preterm compared with unaffected pregnancies but there was no difference in the Congo red false-positive rate according to gestation (Table 3). There was a statistically significantly higher detection rate among the preterm admissions but this may be attributable to most of those cases having severe preeclampsia.

The incidence of preeclampsia is about 2–3 times higher in twins than in singleton pregnancies [11,14] and there was a statistically significantly higher detection rate and false-positive rate among multiple pregnancies. This is probably due to the higher placental mass in twins and triplets [15] since it is believed that misfolded proteins aggregate in the placenta [4,6]. There was also a higher detection rate in multiple pregnancies but this did not reach statistical significance.

A previous pregnancy with preeclampsia is a risk factor in parous women. For example, in one study the incidence of preeclampsia was 42% in those with such a history compared with only 4.9% without [16]. In the current series a similar likelihood ratio was found. However, when the Congo red test performance was compared the observed false-positive rate was considerably higher in those with previous preeclampsia, a highly statistically significant difference (*P*<0.0001). This was an unexpected finding and there is no obvious explanation although it might be related to the high proteinuria incidence in the false-positives. Congo red is associated with proteinuria since it detects the aggregated misfolded proteins, but they are not same.

The previous point-of-care study of Congo red was carried out in the United States in a labour and delivery triage clinic among women who were mostly (66%) White but a sizable minority (29%) were African American [10]. The current study was carried out in China in a complete series of those admitted to hospital for a range of indications including a large number of delivery or symptoms such as bleeding or abdominal pain. More screening studies will be needed in different settings.

Serum PlGF is a well-recognized bio-marker for preeclampsia screening and recommended for first-trimester screening in FIGO guideline [4]. However, the performance for late-onset preeclampsia is rather compromised [17]. CapCord shows promising utility for not only early-onset but also late-onset preeclampsia.

There are at least two potential uses for a late pregnancy Congo red screening test. Firstly, it can be used in a triage situation as with the United States study [10] and in the symptomatic women included in the current study (Table 3). Secondly, it could be a routine test among asymptomatic women. In the current study, whilst the detection rate seemed lower in such women and the false-positive rate higher, these differences were not statistically significant (Table 3). Hence our results support both types of screening.

The interpretation of a non-quantitative test such as this is necessarily subjective. This is particularly so when scoring the Congo red staining pattern into six categories. However, as the study shows, it is possible to reduce the scoring to just two categories. Consequently the new commercial version of the CapCord test includes two control samples which produce positive and negative patterns in addition to the sample being tested.

In conclusion, CapCord is a simple point-of-care urinary misfolded proteins testing device for screening preeclampsia in pregnancy with gestational age after 20 weeks.

## Supporting information

S1 Table Characteristics of women enrolled in the whole cohort. (DOCX)

S1 Data (XLSX)

S2 Data (XLSX)
