# Supplementary material for: Late pregnancy screening for preeclampsia with a urinary point-of-care test for misfolded proteins
Source: PLoS One. 2020 May 20;15(5):e0233214. doi: 10.1371/journal.pone.0233214 (PMC7239432; doi:10.1371/journal.pone.0233214)
Supplement: S1 Table — (DOCX) [file pone.0233214.s001.docx]

**sTable 1 Characteristics of women enrolled in the whole cohort**

| Characteristics | n = 1532 |
| --- | --- |
| Maternal age (years) | 31± 4.7 |
| Multiple pregnancy | 30 (2.0%) |
| Admission reasons |  |
| Symptomatic | 867 (56.6%) |
| Bleeding/discharge /Abdominal pain | 601 (39.2%) |
| Elevated blood pressure | 95 (6.2%) |
| Fetal status/movement | 78 (5.1%) |
| Other | 93 (6.1%) |
| Asymptomatic | 665 (43.4%) |
| Gestational age at sampling (weeks) | 36.6 ± 4.2 |
| 20–23 | 29 (1.9%) |
| 24–27 | 42 (3.9%) |
| 28–33 | 212 (13.9%) |
| 34–36 | 204 (13.3%) |
| 37–38 | 558 (36.4%) |
| ≥39 | 469 (30.6%) |
| Previous preeclampsia | 137 (8.9%) |
| Chronic hypertension | 30 (2.0%) |
| Gestational hypertension | 29 (1.9%) |
| Pre-gestational diabetes | 22 (1.4%) |
| Gestational diabetes | 203 (13.3%) |
| Blood pressure at sampling (mmHG) |  |
| Systolic pressure | 121 ± 15.4 |
| Diastolic pressure | 76.8 ± 11.0 |
| 24 hr urinary protein at sampling* | 0.9 ± 2.6 |

*n=834.
